# Supplementary material for: When noise mitigates bias in human–algorithm decision-making: An agent-based model
Source: PLoS One. 2025 Dec 29;20(12):e0339273. doi: 10.1371/journal.pone.0339273 (PMC12747352; doi:10.1371/journal.pone.0339273)
Supplement: S1 File — In this document we provide additional information about the model specification and calibration. Subsequently, we present several sensitivity analyses that relate to characteristics of the spatial environment in the agent-based model. Finally, we present additional analyses that further probe into some of the results we report in our contribution. (DOCX) [file pone.0339273.s001.docx]

**Supporting Information for**

When noise mitigates bias in human–algorithm decision-making: An agent-based model

In this document, we present background analyses of our agent-based model. We first provide supplementary information about the model specification and calibration (section 1). Subsequently, we present several sensitivity analyses that relate to characteristics of the spatial environment in the agent-based model (section 2). Finally, we present additional analyses that further probe into some of the results we report in our contribution (section 3).

1. Model specification and calibration

1.1. Specification of the spatial environment and model runs

We designed a population-level simulate a temporally bounded spatial agent-based model to environment in which decision-makers randomly interact with subjects. Both decision-makers and subjects navigate a 360° movement space from their initial positions, with movement directionality averaging toward a forward trajectory. The model’s spatial environment consists of a 201 × 201-patch two-dimensional square lattice (based on NetLogo’s internal spatial metric). Additional lattice sizes were evaluated to assess robustness (see Section 2.2). In our model environment, ¾ of the space was designated an interaction zone between residents and decision-makers. In the interaction zone, we used the torus option within NetLogo, which meant the world “wraps” and the subjects and decision-makers to not get stuck at the edges of the environment (1). This option was purposefully introduced to represent a real-world spatial environment. Clearly, residents of a city do not stop when they reach the edge of a police district or neighborhood; they simply continue moving. The remaining ¼ of the model space was designated as the retention zone. Only the subjects in retention go to the retention zone, and they do not move for 100 model ticks (internal NetLogo time measure).

Each model run was calibrated to last for 6000 ticks. Our design goal was that more than 50 percent of the subject population would interact with any of the four decision-makers in a given model run, resulting in an average 620 interactions per model run (62%). This was due to a need for enough interaction data to analyze properly, as well as also allowing for randomness. The desired number of interactions was also a consideration when deciding about the size of the model environment, as a larger environment would lead to fewer interactions, and a smaller environment would lead to more interactions (see Section 2.2).

**1.2. Specification of model parameters**

We consistently varied three parameters throughout our simulations. The first was *advice bias* ($\beta_{A}$) which we restricted to the range of $0\leq$ $\beta_{A}\leq2$. This parameter was informed by the canonical Bayesian model of (un)biased decision-making advice (2). The second parameter we varied was *noise in advice,* which we limited to $-0.5\leq$ $c\leq0.5$. Although we departed in its technical implementation, we were inspired by the use of noisy random walks, modeled as ‘Brownian motion functions’ in policymaking (3). We note that there are many possible ways to model noise within an agent-based modeling environment (4). Random noise in interactions between decision-makers and subjects is another source of noise. A third source of noise we systematically varied as a parameter throughout our simulations is the random distributions of prior beliefs by decision-makers. Table S1 summarizes the parameters used in our model.

Table S1. Parameter description and range

| **Parameter** | **Description** | **Range** |
| --- | --- | --- |
| Bias in advice | Bias in advice received by decision-makers, used to update prior belief | $0\leq$ $\beta_{A}\leq2$. |
| Noise in advice | Noise in advice received by decision-makers, complicated the updating of prior belief | $-0.5\leq$ $c\leq0.5$ |
| Prior beliefs | Distribution or distributions of prior beliefs of decision-makers which are then updated by advice received | $0\leq$ $z_{i}\leq1$ |

**1.3 Model initialization**

At the start of each model run, the $N=1000$ subjects and $N=4$ decision-makers are placed separately in the top ½ of the model environment. The choice of $N=4$decision-makers and $N=1000$ subjects within the model environment are based on data from the United States *Federal Bureau of Investigation* (FBI), which indicates that for cities of 10,000 or fewer residents, there are on average 4.2 police officers per 1000 individuals (5). A randomly selected 10% of the subjects are initiated with a *not bad* ~*B* signifier. In a policing context, this would mean those who are innocent of wrongdoing, could potentially be put in retention (false positive). There are no residents in retention before any decision-maker-subject interactions. The remaining 90% of subjects do not carry this signifier. Therefore, when put in retention, these subjects would be considered *bad* *B* (true positive). How many subjects end up in retention depends on the parameter combination as well as stochastic variation. In retention, it is revealed to the modeler, but not to the decision-maker, whether the resident is *bad* *B* or *not bad* ~*B.* Decision-makers, thus, do not update their priors based on this information. Although positively identified subjects eventually leave retention after 100 ticks, the recording of the true (bad) or false positive (not bad) is carried through to the final analysis.

**1.4 Model simulations**

Each complete run of our model was 6000 ticks (internal NetLogo time step measure). In our simulations, we tested nine levels of ${advisor bias \beta}_{A}=(0, 0.25, 0.5, 0.75, 1, 1.25, 1.5, 1.75, 2).$ Additionally, we also considered a standard uniform prior belief and four additional forms of varied prior beliefs within a normal distribution $z_{i}\mathcal{\sim N}\left( 0.25, 0.50, 0.75, 0.25/0.75), 0 .15 \right)$. We also considered noisy human advice which came with two levels of noise $c=(0.25, 0.5)$, For each of the 135 possible parameter combinations, the model was run 50 times. This meant 6750 total model runs. All data was analyzed after the full model completion (6000 ticks). All simulations were run and controlled using the BehaviorSpace tool included as part of NetLogo 6.3.0, and the data was analyzed in RStudio (6, 1).

**1.5 Calibration of the (biased) normal distributions of prior beliefs**

Our baseline distribution of prior beliefs is a *standard* *uniform distribution* with an expected value of priors to be neutral (EV = 0.50). To obtain distributions of biased (and polarized) prior beliefs we used a *normal distribution*. The symmetrical nature of the normal distribution is attractive as it corresponds with an advice bias $\beta_{A}$ in two directions from its neutral center ($\beta_{A}>1 \wedge\beta_{A} 1)$.

**
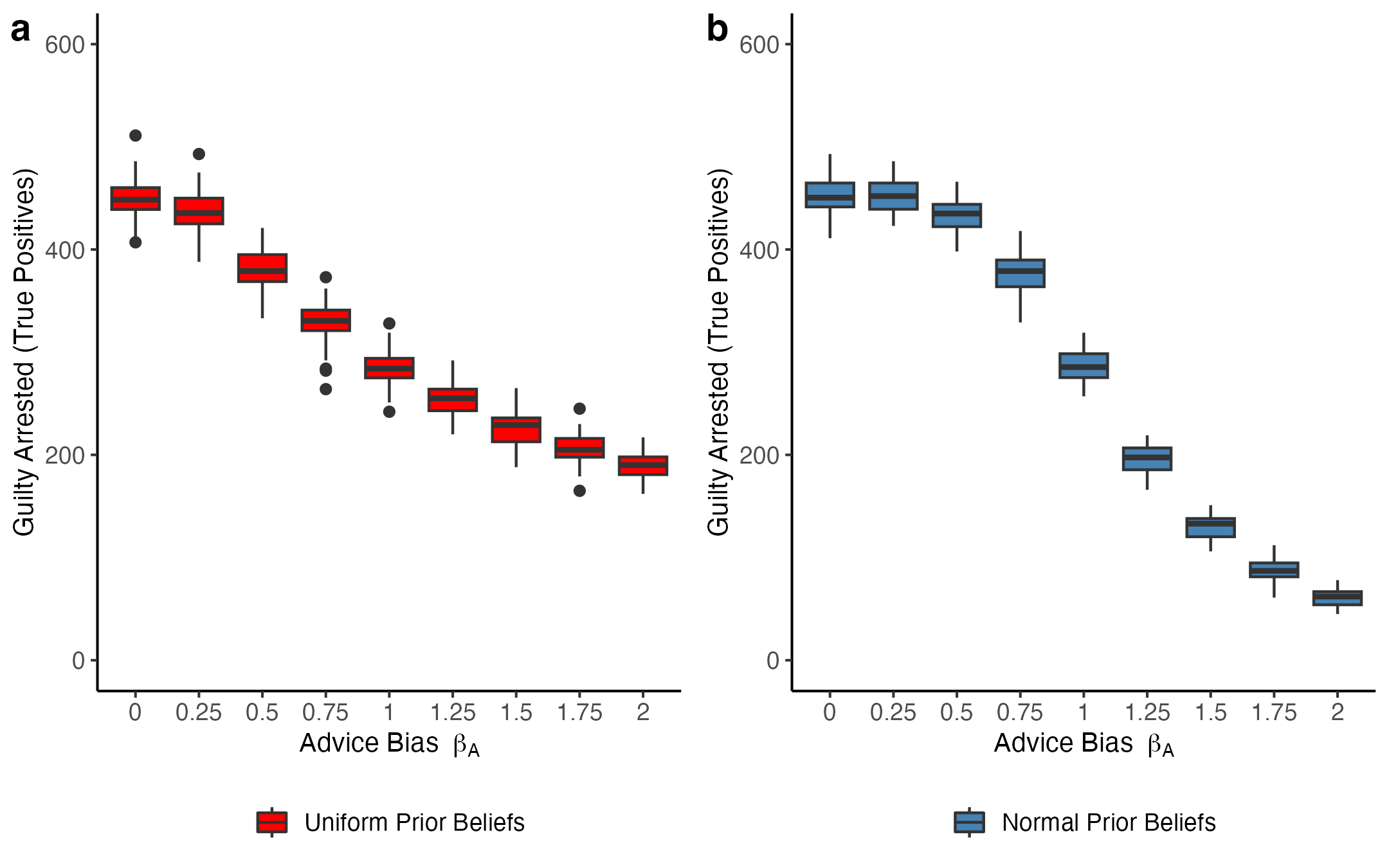
**

**Fig S1. Behavioral outcomes (true positives) of decision-makers under noiseless advice with a uniform distribution of prior beliefs**

(A) Count of true positives across nine levels of advice bias β_(A )for decision-makers with standard uniform prior beliefs (μ = 0). The horizontal line represents the outcomes under uncertainty (β_A = 1). There are 1000 subjects in each model run and 10% are “not bad”. The colored boxes represent the median (line) and the interquartile range (IQR) of 50 simulation runs, holding constant the parameter combinations (β_A, μ) for c = 0. The lines above and below the boxes represent ± 1.5 * IQR. The points above or below the IQR are considered outliers. (B) Count of true positives across nine levels of advice bias β_A, for the model where decision-makers have a neutral normal distribution of prior beliefs〖 z〗_(i )~N(0.5,0.15)

We calibrated the normal distribution of prior beliefs with a mean value of 0.50 to the uniform distribution and obtained $z_{i}\mathcal{\sim N}\left( 0.50, 0.15 \right)$ which is sufficient for our purposes as a neutral distribution (with EV = 0.50). This is demonstrated Figure S1, as the model based on the normal distribution in Fig. 1b responds to advice in all directions similarly (although not exactly) to the baseline model in Fig. 1a which uses the uniform distribution. We then defined three additional normal distributions with biased priors and a standard deviation of 0.15:

1. First a distribution of decision-makers with a bias towards *hesitant* priors was set as: $z_{i \sim}\mathcal{N}\left( 0.25, 0.15 \right)$. Since the decision threshold is 0.5, a lower prior belief value (0$.25)$ corresponds to a lower likelihood of *retention.* (Please note that this is different from advice bias $\beta_{A}$ where values $\beta_{A} < 1$ corresponds with an increased likelihood of *retention*).

2. Secondly, the distribution with a bias towards *aggressive* priors was set as: $z_{i}\mathcal{\sim N}\left( 0.75, 0.15 \right)$.

3. Finally, a bi-modal *heterogenous* or polarized distribution:

$z_{i}\sim0.5\cdot\mathcal{N}\left( \mu_{1},\sigma^{2} \right)+0.5\cdot\mathcal{N}\left( \mu_{2},\sigma^{2} \right)$

which reflects a population of decision-makers with *polarized* prior beliefs, where:

$\mu_{1}= 0.25$ and $\mu_{2}=0.75$ and $\sigma^{2} = 0.15$.

**1.6 Specification of noise in advice**

In our contribution, we noted that when *noise* $c$ increases both the advice bias $\beta_{A}$ and the prior belief $z_{id}$ will have less of an impact on the posterior belief$z_{i}*$. Equation (1) in our contribution presents the central equation of our individual-level model. The random component of that equation is $\varepsilon_{A}\sim(-c,c)$ in which the posterior belief is adjusted by a noise parameter. This noise parameter is a random value between bounds set at $c$ and $-c$, where $0\leq c\leq0.5$. In Figure S2 below we consider a very low value of 0.05. It is important to note that in all three figures, the gray lines are not determinative and are just one possible representation out of infinitely possible random representations under the considered level of noise.

In Figure S2, the gray lines representing the posterior beliefs after both bias and noise are introduced are similar to those in Fig. 1 in our contribution. Until close to the boundary of the domain $z_{i}>0.8$ the lines do not intersect.

In Figure S3 we consider a larger value of noise ($c=0.25$). Unlike Figure S2, there is now an earlier and more consistent intersection between the gray lines, with noise set at a higher level of $c=0.25$. This illustrates that an increased level of noise implies that the transformation between prior and posterior belief is more complex.

The pattern is even more pronounced in Figure S4, where the level of noise is set to of $c=0.50$. In this figure, it is difficult even to track each grey line from start to finish. These are constantly intersecting transformations, moving inside but also outside the model boundaries. Under this higher level of noise, one can find interesting transformations where a decision outcome may profoundly change due to noise. For example, the red dot in the figure is an interesting point, where despite a relatively aggressive prior belief ($z_{i}=\sim0.75$) and neutral advice ($\beta_{A}=1)$, the decision-maker nevertheless decides not to intervene ${(\sim I}_{i})$ due to noise.

Intervene $I_{i}$

Not intervene ~$I_{i}$

$$z_{id}$$

$\beta_{A}=0.5$

$\beta_{A}=1.5$

$$z_{id}*$$

$\beta_{A}=1$

**Fig S2. Individual level model with** $\mathbf{c=0.05}$ **and three different levels of advice bias**

Transformation of prior beliefs into expected utility for different values of bias in advice with noise c = 0.05. Three different levels of bias $\beta_{A}=0.5$, 1, 1.5, were chosen for comparison purposes and are the gray lines that have random variation due to noise. The mid-line is the decision cut-off point between Intervene $I_{i}$ and Not Intervene ~$I_{i}$. The x-axis is the prior belief $z_{i}$ and the y-axis is the transformed posterior belief $z_{i}*$. The blue linear line represents a comparison to $\beta_{A}=$1 and c = 0.05. Our model is bounded between 0 and 1, anything above or below those values is transformed to the nearest endpoint.

**
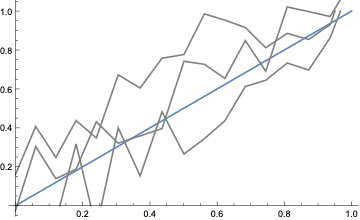
**

Intervene $I_{i}$

Not intervene ~$I_{i}$

$\beta_{A}=0.5$

$\beta_{A}=1.5$

$\beta_{A}=1$

$$z_{id}$$

$$z_{id}*$$

**Fig S3. Individual level model with** $\mathbf{c=0.25}$ **and three different levels of advice bias**

Transformation of prior beliefs into expected utility for different values of bias in advice with noise c = 0.25. Three different levels of bias $\beta_{A}=0.5$, 1, 1.5, were chosen for comparison purposes and are the grey lines that have random variation due to noise. The mid-line is the decision cut-off point between Intervene $I_{i}$ and Not Intervene ~$I_{i}$. The x-axis is the prior belief $z_{i}$ and the y-axis is the transformed posterior belief $z_{i}*$. The blue linear line represents a comparison to $\beta_{A}=$1 and c = 0.05. Our model is bounded between 0 and 1, anything above or below those values is transformed to the nearest endpoint.

**
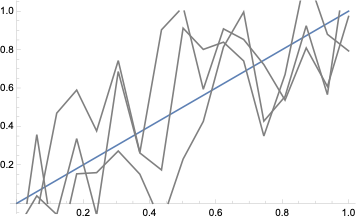
**

$\beta_{A}=1.5$

$\beta_{A}=0.5$

$\beta_{A}=1$

Intervene $I_{i}$

Not intervene ~$I_{i}$

$$z_{id}$$

$$z_{id}*$$

**Fig S4. Individual level model with** $\mathbf{c=0.5}$ **and three different levels of advice bias**

Transformation of prior beliefs into expected utility for different values of bias in advice with noise c = 0.5. Three different levels of bias $\beta_{A}=0.5$, 1, 1.5, were chosen for comparison purposes and are the grey lines that have random variation due to noise. The mid-line is the decision cut-off point between Intervene $I_{i}$ and Not Intervene ~$I_{i}$. The x-axis is the prior belief $z_{i}$ and the y-axis is the transformed posterior belief $z_{i}*$. The blue linear line represents a comparison to $\beta_{A}=$1 and c = 0.05. Our model is bounded between 0 and 1, anything above or below those values is transformed to the nearest endpoint. The red dot is an interesting point in the figure that we discuss in greater detail above.

**1.7 Model availability**

All simulation outputs, scripts used to reproduce the figures, and the full agent-based model code are hosted on the Open Science Framework (OSF). The repository will be accessible at: <https://osf.io/vzmd2>

**2. Sensitivity to characteristics of the spatial environment**

The agent-based model includes a representation of the spatial environment in which decision-makers and subjects ‘wander,’ that is: perform random walks. The encounter of a decision-maker and subject, therefore, depends on chance. This chance can be interpreted as occasion noise in the environment. In this section of the ‘Supplementary Information’ we further explore the sensitivity of the model simulations for assumptions about the spatial environment.

In the first place we explore how occasion noise in the (‘wandering’) spatial environment impacts our simulation results. We do so by implementing a baseline (‘queuing’) model in which we eliminate all noise in the spatial environment – and compare the results of that model with our spatial agent-based model. In Section 2.2 we explore the effects of enlarging or reducing the size of the spatial environment – impacting the rate of decision-makers and subject interaction.

In Section 2.3 we explore the effect of advice bias on interaction rates between decision-makers and subjects. The temporary removal of subjects from the spatial environment would reduce interaction rates especially when advice has an interventionist bias ($\beta_{A}<1$). The lower interaction rates could slightly reduce the effect of interventionist advice bias. When advice has a non-interventionist bias ($\beta_{A}<1$) we would expect the opposite.

**2.1 Noise in the spatial environment and variability in population-level behavior**

Variability in population-level behavior of decision-makers can be attributed to occasion noise in the spatial environment (random walks of decision-makers and subjects). Below, we provide additional analyses that further substantiate these results. What happens when we remove the spatial element of the model entirely? Our agent-based model is particularly relevant in contexts such as police officers patrolling neighborhoods. Such a spatial environment is conducive to occasion noise, as we argue in our contribution. Other spatial contexts of decision-makers checking subjects take the form of controlled environments. For example, border patrol agents checking passports of passengers queued in line, or medical professionals examining patients or scans to make a diagnosis, operate in environments that constrain movement and eliminate occasion noise

To verify the effect of eliminating noise in the spatial environment, we implemented a simple simulation model in RStudio (6). This simple simulation model is based on a series of repeated interactions between a fixed set of four decision-makers processing a pool of subjects. Notably, this is a queuing model, devoid of spatial dynamics, designed to contrast with our agent-based model, which includes wandering agents in a spatial environment. We simulated the baseline model from Equation (1) in our contribution, drawing upon the random uniform distribution of prior beliefs. We set noise in advice to zero (*c* = 0) to simplify the model and rule out any other source of noise at the individual level other than the uniform random distribution of priors.

Figure S5 presents the results of the analyses and compares these results with those from the spatial agent-based model with the same parameter specification. The figure clearly shows that the size of the interquartile range boxes is smaller for the *non-spatial* queuing model, throughout the range of advice bias, than for the agent-based *spatial* model. Moreover, Figure S5 also reveals that under interventionist advice ($\beta_{A}$ < 1) the activity of decision-makers (guilty arrested) is also higher in the non-spatial queuing model than in the spatial agent-based model.

**
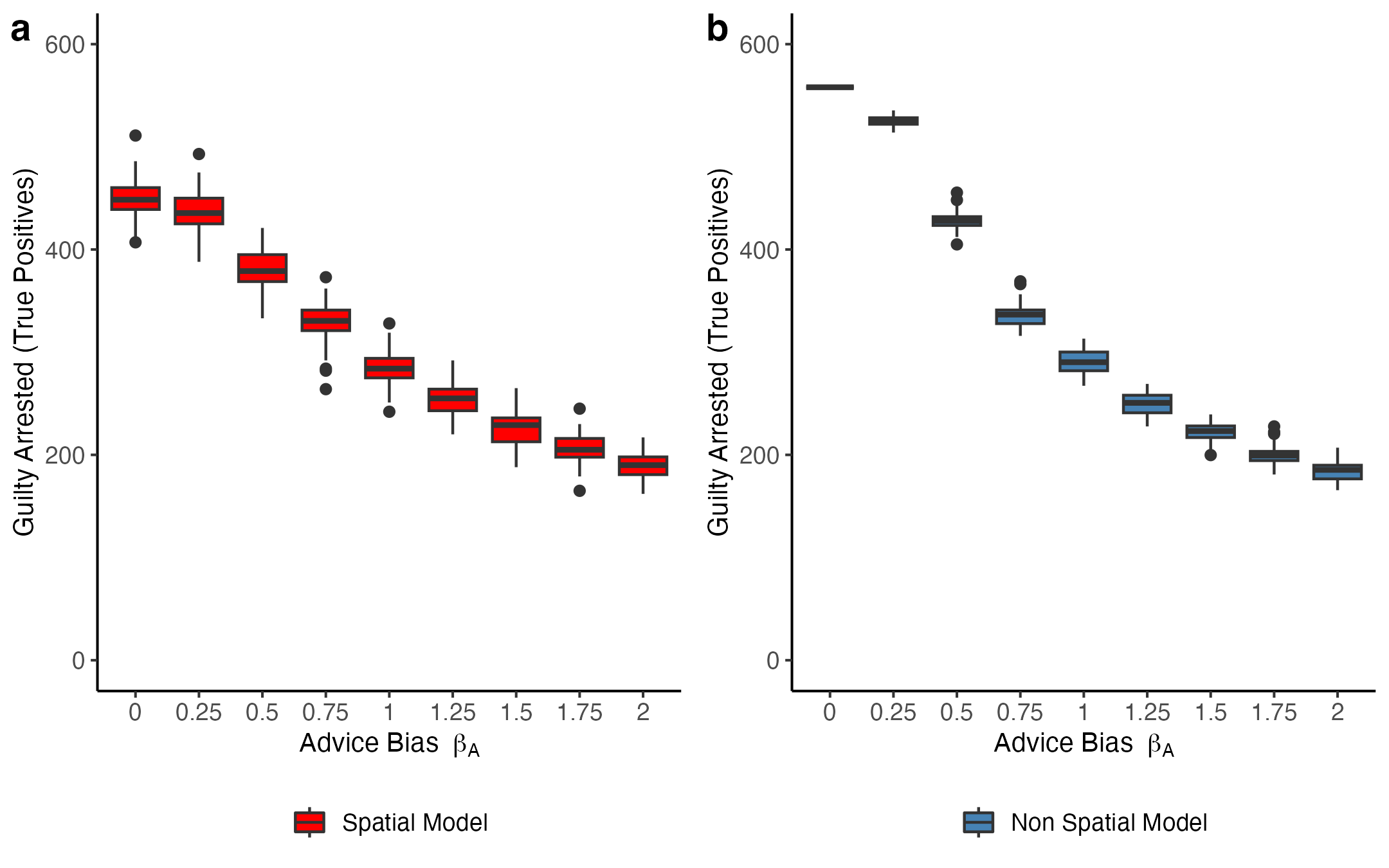
**

**Fig. S5. Behavioral outcomes of decision-makers under noiseless (algorithmic) advice for spatial and non-spatial models**

(A) Count of true positives across nine levels of advice bias β_A for the spatial model. The horizontal line represents the outcomes under uncertainty (β_A = 1, μ = 0). There are 1000 subjects in each model run and 10% are not bad, while the rest are bad. The colored boxes represent the median (line) and the interquartile range (IQR) of 50 simulation runs, holding constant the parameter combinations (β_A, μ) for c = 0. The lines above and below the boxes represent ± 1.5 * IQR. The points above or below the IQR are considered outliers. (B) Same plot and model details except that the environment is non-spatial.

Table S2 shows that the standard deviation is consistently larger for the spatial agent-based wandering model than for the non-spatial queuing model. Thus, we verified that noise in the environment, a form of occasion noise, leads to increased variation in population-level behavioral outcomes. However, the effect of controlling the environment is especially large when advice bias is interventionist ($\beta_{A}$ < 1).

**Table S2. Standard deviation of guilty residents arrested (true positives) for agent-based and simulation models**

| **Advice Bias** | **‘Wandering’**  **environment** | **‘Queuing’**  **environment** |
| --- | --- | --- |
| 0 | 18.6 | 0 |
| 0.25 | 18.3 | 5.41 |
| 0.5 | 17.8 | 9.9 |
| 0.75 | 18.4 | 12.1 |
| 1 | 16.1 | 10.2 |
| 1.25 | 15.6 | 12.7 |
| 1.5 | 14.9 | 10.4 |
| 1.75 | 13.5 | 12.9 |
| 2 | 12.6 | 10.7 |

This table reports the standard deviation of the number of guilty residents arrested (true positives) under two model environments: the *wandering* spatial model and the *queuing* non-spatial model, across increasing values of advice bias (βₐ). The results show that variability in arrests is consistently higher in the wandering model compared to the queuing model. However, the difference in standard deviation between the two environments decreases as βₐ approaches 2. At βₐ = 0, there is no variation in the queuing model because advice is operationalized as a “command,” meaning no randomness is introduced through environmental interaction. As βₐ increases, the probabilistic element of the decision-making process introduces noise into the queuing non-spatial model, resulting in greater variability and a convergence toward the wandering model’s levels of dispersion.

.

**2.2. Size of the spatial environment**

In our agent-based model simulations, the spatial environment in which decision-makers and subjects perform their random walks is composed of a 201 × 201 grid of patches. Each patch is a square piece of model ground, where movement and interactions among agents take place (1). Patches are akin to the “squares that make up a chessboard” (7). The size of the spatial environment affects the likelihood that decision-makers and subjects encounter each other in any given number of time-steps. This likelihood, in turn, may affect the number of residents put in retention.

To verify whether our simulation results are sensitive to the size of the environment, we performed additional simulations in a smaller and a larger environment. One environment doubled the dimensions (401 x 401 patches), of our standard agent-based model (201 x 201 patches), while another halved them (101 x 101 patches). We simulated the baseline model from Equation (1) in our contribution, drawing upon the random uniform distribution of prior beliefs. We set noise in advice to zero (*c* = 0).

The results of these simulations are presented in Figure S6. These results demonstrate the consequences of more interactions between decision-makers and subjects in a smaller spatial environment. In the smaller spatial environment, more arrests are made. In the larger spatial environment, fewer arrests are made. The pattern of decision-makers’ activity (guilty arrested) as a function of advice bias $\beta_{A}$is comparable for the three spatial environments that differ in size. Hence, we conclude that our results are robust under different sizes of the modeled spatial environment.

**
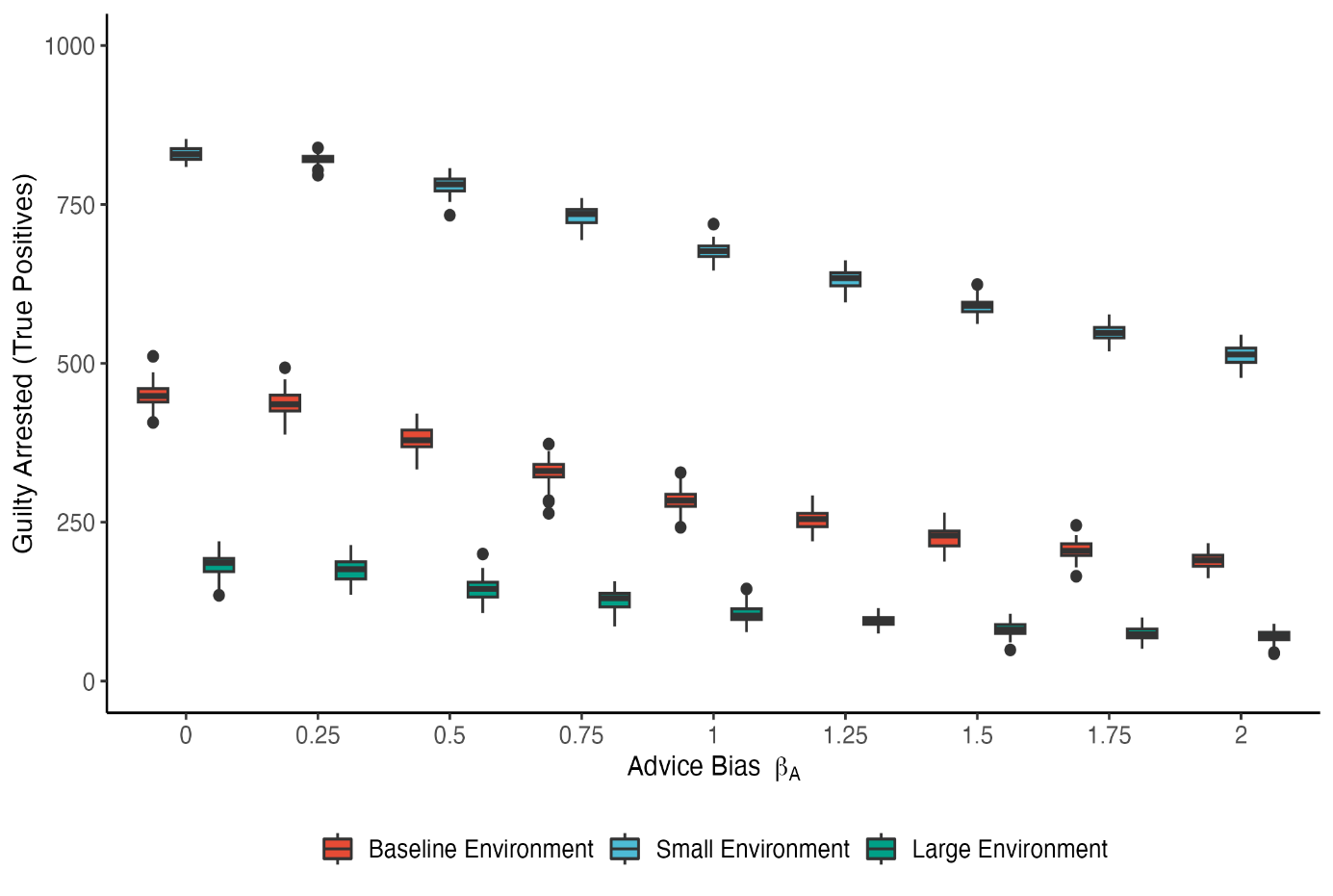
**

**Fig S6. Behavioral outcomes of decision-makers under noiseless (algorithmic) advice and different model environment sizes**

Count of true positives across nine levels of advice bias $\beta_{A}$. The horizontal line represents the outcomes under uncertainty ($\beta_{A}$ = 1, *μ* = 0). The colored boxes represent the median (line) and the interquartile range (IQR) of 50 simulation runs, holding constant the parameter combinations , *μ*) for *c =* 0. There are 1000 subjects in each model run and % are not bad, while the rest are bad. The lines above and below the boxes represent $\pm$ 1.5 * IQR. The points above or below the IQR are considered outliers. Colors represent different model environment “patch” sizes, Baseline Environment (201 x 201), Large Environment (401 x 401), and Small Environment (101 x 101).

**2.3. Rate of interaction under different values of advice bias**

Putting subjects in retention temporarily removes subjects from the spatial environment. Such an act would reduce interaction rates between decision-maker and subjects; especially when advice is biased towards interventionist ($\beta_{A}<1$). Then, decision-makers will encounter fewer subjects even though they are more interventionist due to the advice. Conversely, when advice is biased towards non-interventionist ($\beta_{A}>1$), decision-makers will put less subjects in retention, leaving more subjects in the spatial environment, which increases the interaction rate. This increased interaction rate would reduce the effect of non-interventionist advice.

To explore these countervailing effects in our model simulations, we developed an analytical baseline model. The analytical model departs from the assumption that decision-makers draw their priors from the uniform random distribution of prior beliefs specified in Equation (1) in our contribution. Given the random uniform distribution, with an expected value EV = 0.5, there is a clear relation between the correct and false categories of subjects. The relation is defined by a convex combination of the expected value of the prior (with *μ* =.5), the proportion of bad subjects in the population (*π*_B_), and advice bias (*β_A_*).

- The expected number of true positives: *E*(*TP*) = *π*_B_ $\cdot z_{id}^{\beta_{A}}$ *N.*
- The expected number of false positives: *E*(*FP*) = (1-*π*_B_)$z_{id}^{\beta_{A}}$ *N.*
- The expected number of false negatives: *E*(*FN*) = *π*_B_$(1-z_{id}^{\beta_{A}})$ *N.*

With the proportion of bad subjects set to our model parameters at *π*_s_ = .9 and *N* = 1,000, we can easily verify that under noiseless advice *E*(*FP*) = ${.5}^{\beta_{A}}\cdot$ 100; *E*(*FN*) = *E*(*FP*) = ($1-{.5}^{\beta_{A}})\cdot$ 900; and *E*(*TP*) = ${.5}^{\beta_{A}}\cdot$.900. Of course, in our population-level agent-based model, not every subject has an interaction with a decision-maker, so *N* $\neq$ 1,000. Suppose we pick a value close to the actual number of interactions in our agent-based model that result in a true positive (*N* = 550). Then we should expect the analytical model to approach the results of our population-level agent-based model – particularly, for values of advice bias close to neutral (*β_A_* =1). The number of true positives in that case would be ½ $\cdot$ 550 = 275.

**
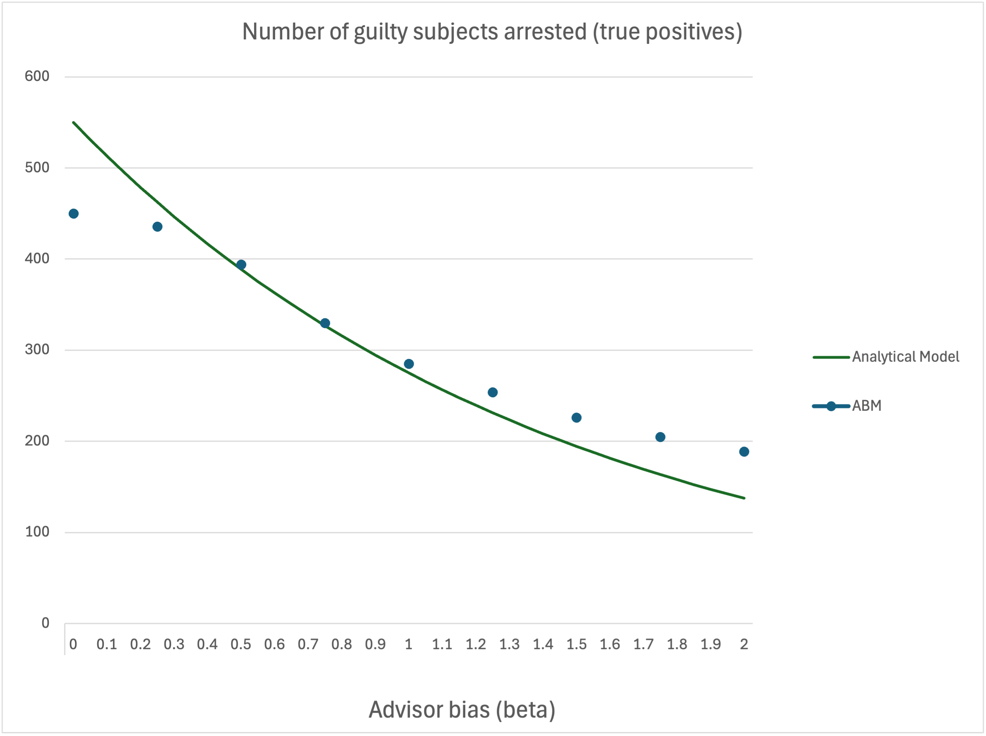
**

**Fig S7. Behavioral outcomes of decision-makers under noiseless (algorithmic) advice for analytical and population-level (ABM) models**

The mean true positive counts for the analytical model. For the analytical model, these counts are computed for every 0.05 value between 0 and 2, then smoothed into a line. For the population-level agent-based model ABM, the mean true positive counts were plotted as blue dots. These were plotted for the standard nine levels of advice bias βA.

Figure S7 presents the number of bad subjects put in retention in our agent-based model for each of the nine values of advice bias *β_A_*. In addition, this figure plots the curve ${275}^{\beta_{A}}$ as a function of advice bias *β_A_*. Figure S7 shows that the difference in population-level behavior due to the removal of subjects in retention is not large, but noticeable. We can verify that, as expected, the number of true positives in the analytical function is slightly higher than in the agent-based model for advice bias 0 < *β_A_* < 1. We also verify that the number of true positives in the analytical function is slightly lower than in the agent-based model for advice bias *β_A_* > 1.

The relationship between advice bias and interaction rates is further confirmed in Figure S8, which presents the interaction rates in the agent-based model as a function of advice bias. When advice bias $\beta_{A}=0$, there are only 518 total interactions. When $\beta_{A}=2$, there are 718 interactions, a 43 percent increase.

**
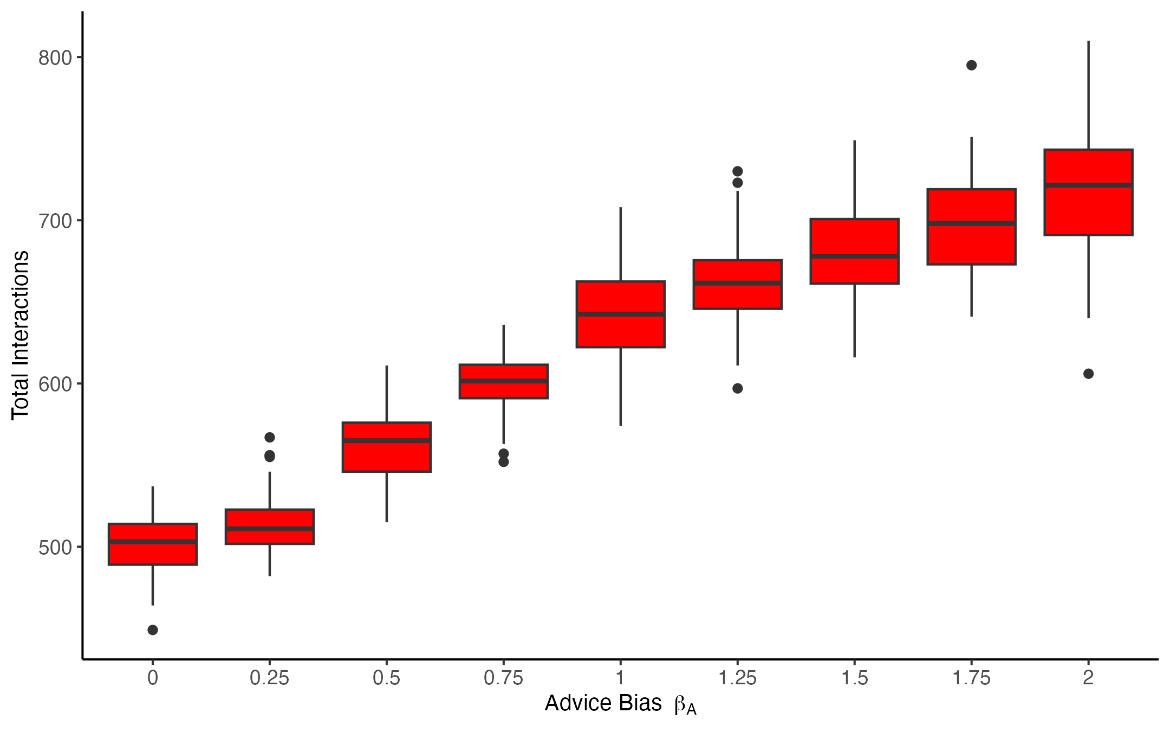
**

**Fig S8. Total interactions of decision-makers under noiseless (algorithmic) advice with standard uniform prior beliefs**

Average total interactions across nine levels of advice bias $\beta_{A}$ for the agent-based model. Each average is across the 50 simulation runs for that level of advice bias. The boxes represent the median (line) and the interquartile range (IQR) of 50 simulation runs, holding constant the parameter combinations ($\beta_{A}$, *μ*) for *c =* 0. There are 1000 subjects in each model run. The lines above and below the boxes represent $\pm$ 1.5 * IQR. Total interactions include all of the decision-maker, subject interactions during each model run.

**3. Additional Analyses**

**3.1 The plateau effect of the polarized distribution**

An unexpected finding from our model is the unique pattern of behavioral outcomes when decision-makers have *polarized* prior beliefs (see Fig. 4 in main document). When advice bias $\beta_{A}\geq$ 1, the advice bias does not affect the behavior of the polarized population. The numbers remain on a plateau, which is comparable to full uncertainty$\beta_{A}=$ 1. We find variations of this effect under conditions of noise as well (see Fig. 5 in our contribution).

Since the possible range of values of $\beta_{A}$is between [0, ∞ > we need to verify whether the plateau effect still can be observed for relatively high values of $\beta_{A}$. To check whether this is that case, we ran an additional polarized model including values of$\beta_{A}=5, \beta_{A}=10 and \beta_{A}=100$. The results are shown below in Figure S9. The pattern continues even with these high levels of bias in advice, further providing support that a genuine plateau effect exists at the full range of values of advice bias.

**
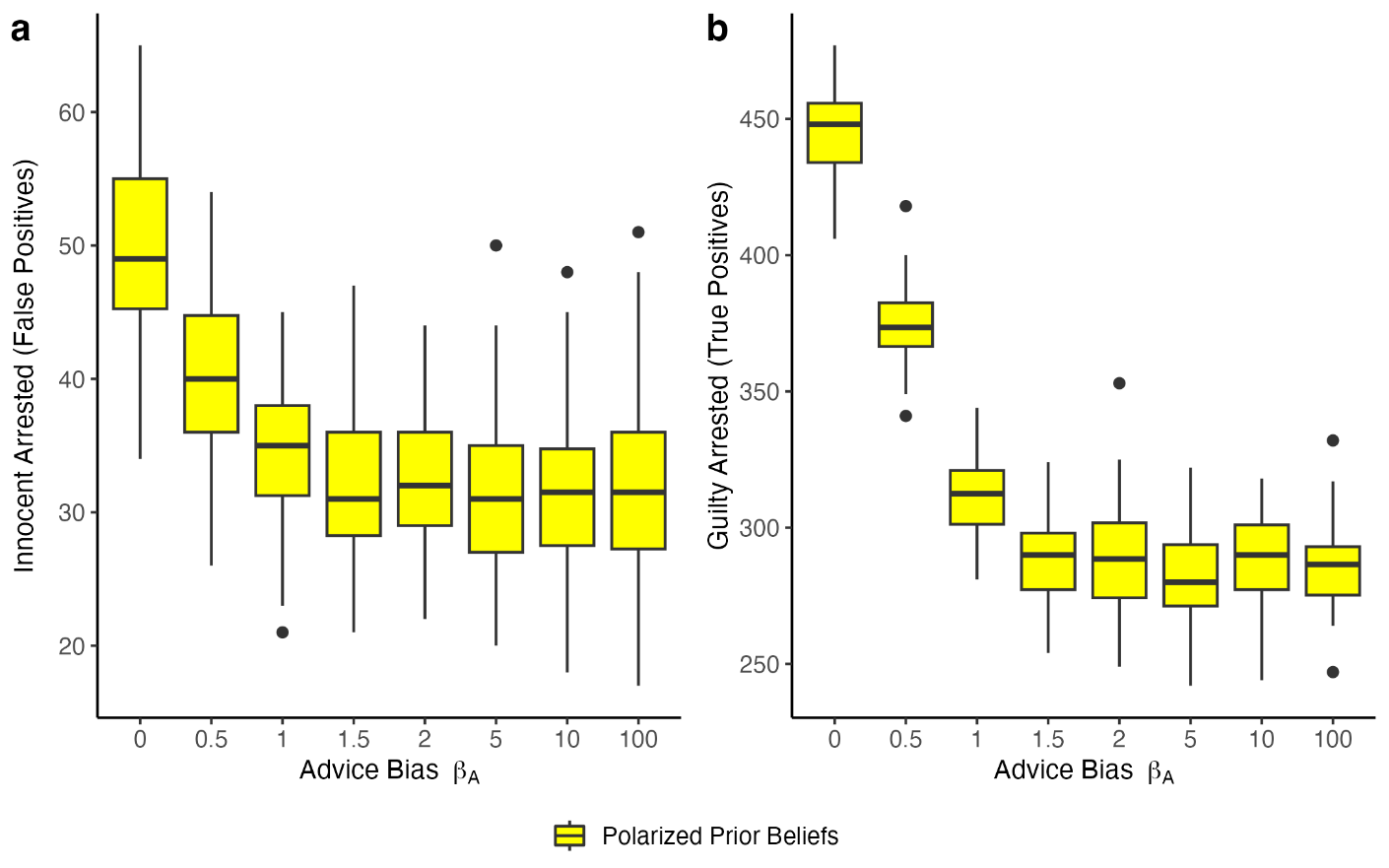
**

$$z_{i}$$

**Fig S9. Behavioral outcomes of polarized populations of decision-makers under noiseless (algorithmic) advice with higher levels of bias**

(A) Count of true positives across eight evels of advice bias $\beta_{A}$ which go up to a value of 100. The horizontal line represents the outcomes under uncertainty ($\beta_{A}$ = 1, *μ* = 0). The colored boxes represent the median (line) and the interquartile range (IQR) of 50 simulation runs, holding constant the parameter combinations ($\beta_{A}$, *μ*) for *c =* 0. The lines above and below the boxes represent $\pm$ 1.5 * IQR. The points above or below the IQR are considered outliers. (B) Same plot except for the behavioral outcome which is the count of false positives across nine levels of advice bias $\beta_{A}$*.*

In the results section of our contribution, we report the plateau effects as a pattern of population-level behavior. This pattern emerges organically and is not the result of individual-level decision-maker differences. Advice bias $\beta_{A}$affects the subject's individual level characteristic value $z_{id}$ through an exponential transformation $z_{id}^{\beta_{A}}$. The resulting value functions as a (possibly biased) random draw during each interaction. Similarly, biased prior beliefs are modeled as a random draw during each decision-maker interaction $z_{i}\sim\mathcal{N}\left( \mu_{1},\sigma^{2} \right)$. Thus, there are no fixed differences between decision-makers. In our model, the polarized distribution defines each subject–decision-maker interaction as having a 50% probability of drawing from either the aggressive or the hesitant distribution $z_{i}\sim0.5\cdot\mathcal{N}\left( 0.25, 0.15 \right)+0.5\cdot\mathcal{N}\left( 0.75, 0.15 \right)$.

**3.2 Reversion to prior beliefs under a moderate level of advice noise**

One of the results of our simulations is that noise in advice pushes the behavioral outcomes towards the uncertain results, that is more similar to $\beta_{A}$ = 1. Noise in advice, thus, makes decision-makers revert to their prior beliefs. Fig. 5 in the results section of our contribution compares effects of noiseless (algorithmic) advice ($c=0$) with those of very noisy (human) advice ($c=0.50$). To save space we did not report the simulation results in our contribution for moderately noisy advice ($c=0.25).$ Figure S10 presents the results of this simulation (behavior of decision-makers with various distributions of prior beliefs under moderate noise in advice). The results presented in Figure S10 reveal a similar, though less pronounced, pattern as Figure 5b in our contribution. This shows that advice noise has a gradual effect on reversion to prior beliefs.


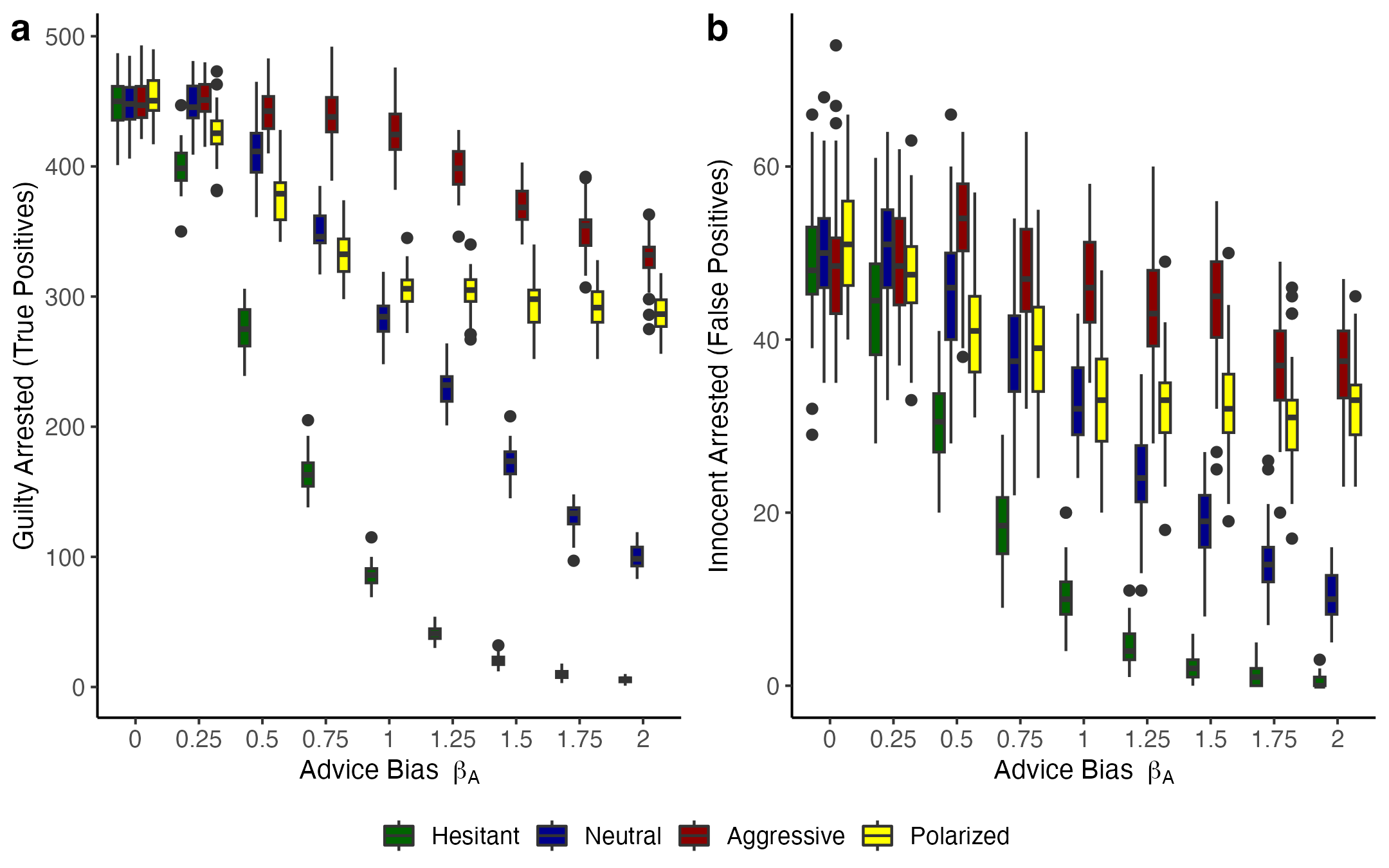


Fig S10. Behavioral outcomes of biased populations of decision-makers under noisy (human) advice with different levels of bias

(A) Count of true positives across nine levels of advice bias $\boldsymbol{\beta}_{\boldsymbol{A}}$. The horizontal line represents the outcomes under uncertainty ($\boldsymbol{\beta}_{\boldsymbol{A}}$ = 1, *μ* = 0). The colored boxes represent the median (line) and the interquartile range (IQR) of 50 simulation runs, holding constant the parameter combinations ($\boldsymbol{\beta}_{\boldsymbol{A}}$, *μ*) for *c =* 0.25. The lines above and below the boxes represent $\boldsymbol{\pm}$ 1.5 * IQR. The points above or below the IQR are considered outliers. Colors represent different distributions of priors in the population of decision-makers (hesitant: *μ* =0.25; uncertain: *μ* = 0; aggressive: *μ* =0.75; polarized: *μ*_1_ =0.25, μ2 =0.75). Other than the counts as behavioral outcomes (and therefore the range), the two plots are constructed using the same approach. (B) Same plot except for the behavioral outcome which is the count of false positives across nine levels of advice bias $\boldsymbol{\beta}_{\boldsymbol{A}}$.

**3.3 Noise in advice and variability in population-level behavior**

Our simulations reveal that advice noise does not lead to increased variability in population-level behavior of decision-makers. In our contribution we base this conclusion on a visual inspection of the boxplots in the figures for noiseless advice (*c* = 0), a moderate level of noise in advice ($c=$.25) and a high level noise in advice ($c=$.50). These boxplots do not visually increase in size for larger values of *c*, indicating that variability in population-level behavior of decision-makers does not depend on advice noise.

Figure S11 presents a more structured comparison of variability in population-level behavior of decision-makers under different levels of advice noise. We simulated the baseline model from Equation (1) in our contribution, drawing upon the random uniform distribution of prior beliefs, for the nine values of advice bias ($\beta_{A}$). We varied noise in advice across the three levels (*c* = 0; 0.25; 0.5).

**
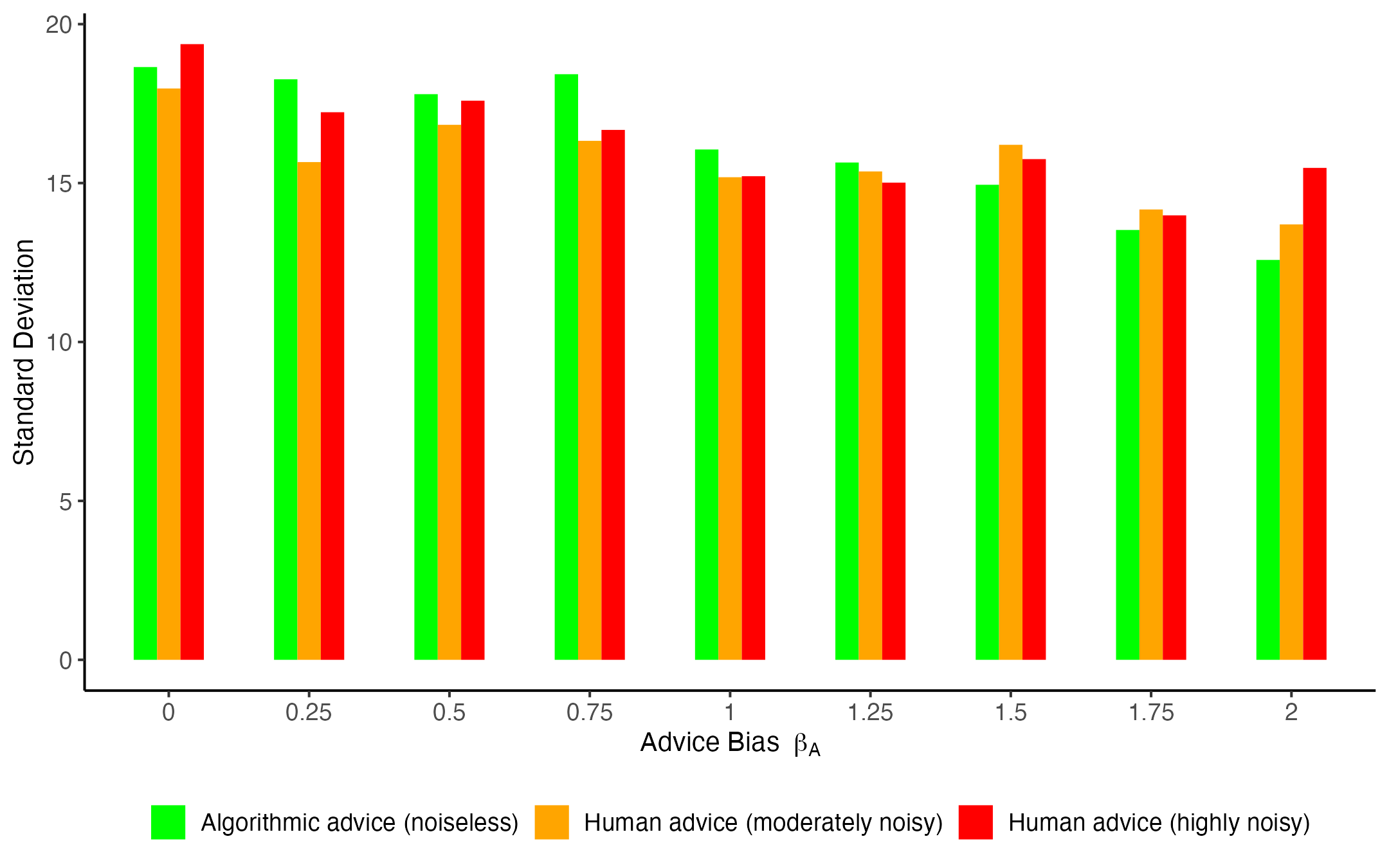
**

**Fig S11. Standard deviation of behavioral outcomes of decision-makers under noiseless and noisy advice**

Average standard deviation values of true positives across nine levels of advice bias $\beta_{A}$ for the agent-based model. Each average is across the 50 simulation runs for that level of advice bias. Colors represent different levels of ambiguity in advice (Algorithmic advice (noiseless): *𝑐* = 0; Human advice (moderately noisy) $c = 0.25$; Human advice (highly noisy) $c = 0.5$).

Inspection of Figure S11 shows an increase in advice noise does not lead to consistently higher standard deviations. Only at the high end of the spectrum of advice bias (towards non-interventionist; $\beta_{A}=2$) we observe slightly larger standard deviations in population-level behavior when advice noise increases. However, these differences are relatively small. In our contribution, we explain the lack of an association between advice noise and variability in population-level behavior results from the symmetrical nature of advice noise, which leads to random variations at the individual level canceling out at the population level. Individual noise canceling out at the population level has been proposed by others (8-9).

**3.4 Modifying the proportion of bad and not bad subjects in the population**

As noted in the main contribution, the designation of subjects in our model as "bad" or "not bad" affects only the distribution of true and false positives, without influencing the combined retention total (sum of true and false positives). That is, these labels serve purely as evaluative benchmarks and do not impact behavior within the model itself. To further illustrate this point, we constructed a variant of our model in which the proportion of subjects labeled as "bad" and "not bad" is reversed: now 10% of the population is labeled as "bad" and 90% as "not bad."

As shown in Figure S12, the results of this flipped model mirror those of the original: approximately 90% of the combined retention total in the original model consisted of true positives, this figure drops to approximately 10% in the flipped model. Correspondingly, false positives now account for the remaining ~90%. These results demonstrate that the behavioral outcomes are functionally inverted when the underlying evaluative labels are reversed. Aside from minor stochastic variation due to random noise, the behavioral dynamics across the two models are nearly symmetrical, reinforcing our assertion that the "bad"/"not bad" labels influence only the post hoc evaluation of outcomes, not the model's internal dynamics.

**
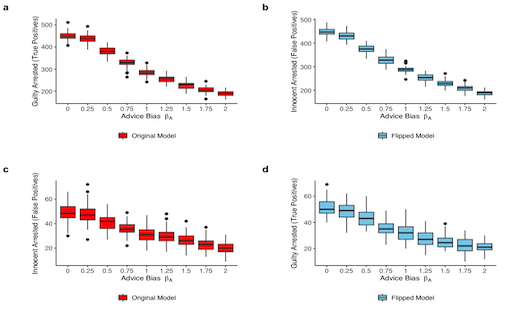
**

**Fig S12. Behavioral outcomes of decision-makers under noiseless (algorithmic) advice for original and “flipped” model**

(A) Count of true positives across nine levels of advice bias $\beta_{A}$ for the original model. There are 1000 subjects in each model run and 10% are not bad, while the rest are bad, in the original model. The colored boxes represent the median (line) and the interquartile range (IQR) of 50 simulation runs, holding constant the parameter combinations ($\beta_{A}$, *μ*) for *c =* 0. The lines above and below the boxes represent $\pm$ 1.5 * IQR. The points above or below the IQR are considered outliers. (B) Same plot detail but the labels are flipped: 10% of subjects are designated as bad and the remaining 90% as not bad, the behavioral outcome is false positives. (C) Same plot and original model details with the behavioral outcome being false positives. (D) Same plot detail but the labels are flipped: 10% of subjects are designated as bad and the remaining 90% as not bad, the behavioral outcome is true positives

**SI References**

1. U. Wilensky, NetLogo (Center for Connected Learning and Computer-Based Modeling, Northwestern Univ., Evanston, IL, 1999). <http://ccl.northwestern.edu/netlogo/>
2. R. L. Calvert, The value of biased information: A rational choice model of political advice. J. Polit. 47, 530–555 (1985). <https://doi.org/10.2307/2130895>
3. S. Callander, Searching for good policies. Am. Polit. Sci. Rev. 105, 643–662 (2011).
4. P. Steiglechner, M. A. Keijzer, P. E. Smaldino, D. Moser, A. Merico, Noise and opinion dynamics: How ambiguity promotes pro-majority consensus in the presence of confirmation bias. R. Soc. Open Sci. 11, 231071 (2024). [https://doi.org/10.1098/rsos.231071](http://ccl.northwestern.edu/netlogo/)
5. FBI, Crime in the United States, 2019. <https://ucr.fbi.gov/crime-in-the-u.s/2019/crime-in-the-u.s.-2019/tables/table-71/table-71.xls>
6. RStudio Team, RStudio: Integrated development for R (RStudio, PBC, Boston, MA, 2020). <http://www.rstudio.com/>
7. Center for Connected Learning & Computer-Based Modeling, How to use the patches primitive in NetLogo? Beginner’s Interactive NetLogo Dictionary (2021). [https://ccl.northwestern.edu/netlogo/bind/primitive/patches.html](http://ccl.northwestern.edu/netlogo/)
8. M. Hilbert, Toward a synthesis of cognitive biases: How noisy information processing can bias human decision making. Psychol. Bull. 138, 211–237 (2012). [https://doi.org/10.1037/a0025940](http://ccl.northwestern.edu/netlogo/)
9. C. R. Sunstein, Governing by algorithm?: No noise and (potentially) less bias. Duke Law J. 71, 1175–1205 (2022).
