# Supplementary material for: When noise mitigates bias in human–algorithm decision-making: An agent-based model
Source: PLoS One. 2025 Dec 29;20(12):e0339273. doi: 10.1371/journal.pone.0339273 (PMC12747352; doi:10.1371/journal.pone.0339273)
Supplement: Attachment — Submitted filename: Respone to Reviewers.docx [file pone.0339273.s003.docx]

**PLOS ONE
Review Response**

**To:** PLOS ONE Reviewers and Editor
**Subject:** Review Response for Manuscript “When Noise Mitigates Bias in Human–Algorithm Decision-Making: An Agent-Based Model”

Dear Reviewers and Editor

Thank you for the thoughtful consideration and reviews of our manuscript, *“When Noise Mitigates Bias in Human–Algorithm Decision-Making: An Agent-Based Model,”* submitted for consideration as a Research Article in *PLOS ONE.*

Reviewer One provided two excellent points of feedback. The first concerned the number of “bad” individuals in the model simulations and why this does not influence model dynamics. We expanded on this in the manuscript on page 8, providing additional clarification on our decision to reduce complexity in order to isolate key mechanisms. Additionally, we offered guidance on page 18 regarding how future research could incorporate a trait-based approach.

The second point addressed the asymmetrical behavioral patterns observed in polarized groups. To strengthen this section, we added a new example concerning the politics of immigration, another highly polarized issue, that follows a similar pattern to the one established in our model.

Reviewer Two directed us to a critical body of literature relevant to our manuscript, including studies on the potential empirical benefits of noise in decision-making. Throughout the introduction and discussion, we incorporated this relevant scholarship. These additions improved the manuscript by situating our research within the emerging literature on the benefits of noise in decision-making.

We also addressed all journal and editorial requirements and suggestions mentioned in the review. These, along with all reviewer comments, were invaluable in improving the manuscript. Below, we detail our responses to each comment and suggestion, and indicate where in the manuscript we made the corresponding revisions. Please see the table summarizing our changes on the next page.

Thank you again for your time and consideration. We look forward to the possibility of contributing this work to *PLOS ONE.*

Sincerely,
**Spencer Poodiack Parsons**
University of Twente
s.j.poodiack-parsons@utwente.nl
Tel: +31 681355961

**Prof. dr. René Torenvlied**
University of Twente
r.torenvlied@utwente.nl
Tel: +31 534892825

| **Comment** | **Response** | **Location in MS.** |
| --- | --- | --- |
| **Reviewer #1 Comments** |  |  |
| R1.1) Better motivate the statement that the percentage of bad individuals does not influence the model dynamics (around line 180). | JR1) Ensure manuscript meets PLOS ONE style, formatting, and file-naming requirements. | We have adjusted our file naming and formatting to match PLOS One requirements. |
| R1.2) Add at least one more example and deeper interpretation of behavioral asymmetry (around line 383). | We added a discussion of immigration politics which demonstrates similar dynamics. We included this both on P. 16 where recommended and again on P. 20. | P.16 and P. 20 |
| **Reviewer #2 Comments** |  |  |
| R2.1) Engage with and cite empirical studies on noise in human judgment and behavior (references provided). | The addition of the reccomended studies (and additional related scholarship) have improved the manuscript and have been included throughout the Introduction and Discussion sections. | Throughout Introduction/Discussison |
| **Additional Editor Requests** |  |  |
| E1) Clarify why the percentage of “bad individuals” does not affect model dynamics. | I have added additional clarification on P. 8 while also considering a potential extention to this approach for future research on P. 18. | P. 8 and P. 18 |
| E2) Expand on asymmetry in emergent behavioral patterns with at least one additional example and interpretation. | In the revised manuscript, we added a new example from U.S. immigration politics to demonstrate how asymmetric responsiveness operates outside of the health domain. | P. 16 and P.20 |
| E3) Situate the theoretical model within broader empirical literature on noise in human decision-making (as suggested by Reviewer #2). | These addition of these studies (and additional related scholarship) have improved the manuscfript and have been included throughout the Introduction and Discussion sections. | Throughout Introduction/Discussison |
| **Journal / Editorial Requirements** |  |  |
| JR2) Ensure code is made available per PLOS ONE open-code policies. | We have created an OSF repository containing the agent-based model as well as the data and scritps used our manuscript. |  |
| JR3) Update the Data Availability Statement to identify the data repository and ensure free public access upon publication. | We have updated the data availability statement to point towards our new OSF repository, which we will make public prompltly if we are accepted by PLOS One. | P. 27 |
| JR4) Upload Figure 3c or remove references to it in the manuscript text. | The reference to this figure has been removed from the text, it did not enhance the manuscript arguments. | P. 9 |
| JR5) Include captions for all Supporting Information files and update in-text citations to match. | Removed the figure which was inserted into Table S2. It was not necessary for the analysis. All figures are captioned. | S.I.P. 29 |
| JR6) Evaluate reviewer-recommended citations and include if relevant. | Very suseful suggestion, added these studies throughout the manuscript |  |
| JR7) Review references for completeness, accuracy, and retraction status; revise or justify as needed. | Fixed any issues uncovered with refrences. Added refrences for new materials. | P. 27-30 |
| JR8) To ensure your figures meet our technical requirements, please review our figure guidelines: | Used the recommended PLOS One NAAS figure tool to update figures and confiurm requirements are met. |  |
